# Venous thromboembolism in Croatia – Croatian Cooperative Group for Hematologic Diseases (CROHEM) study

**DOI:** 10.3325/cmj.2015.56.550

**Published:** 2015-12

**Authors:** Dražen Pulanić, Velka Gverić-Krečak, Zlatka Nemet-Lojan, Hrvoje Holik, Božena Coha, Renata Babok-Flegarić, Mili Komljenović, Dijana Knežević, Mladen Petrovečki, Silva Zupančić Šalek, Boris Labar, Damir Nemet

**Affiliations:** 1 Division of Hematology, Department of Internal Medicine, University Hospital Center Zagreb, Zagreb, Croatia; 2 Medical School University of Zagreb, Zagreb, Croatia; 3 Faculty of Medicine Osijek, J.J. Strossmayer University of Osijek, Osijek, Croatia; 4 Division of Internal Medicine, General Hospital Šibenik, Šibenik, Croatia; 5 Division of Internal Medicine, General Hospital “Dr. Tomislav Bardek,” Koprivnica, Croatia; 6 Division of Internal Medicine, General Hospital “Dr. Josip Benčević,” Slavonski Brod, Croatia; 7 Division of Internal Medicine, General Hospital Varaždin, Varaždin, Croatia; 8 Division of Internal Medicine, General Hospital “Hrvatski Ponos,” Knin, Croatia; 9 Department of Clinical Laboratory Diagnosis, Dubrava University Hospital, Zagreb, Croatia; 10 Department of Medical Informatics, Rijeka University School of Medicine, Rijeka, Croatia

## Abstract

**Aim:**

To analyze the incidence and characteristics of venous thromboembolism (VTE) in Croatia.

**Methods:**

The Croatian Cooperative Group for Hematologic Diseases conducted an observational non-interventional study in 2011. Medical records of patients with newly diagnosed VTE hospitalized in general hospitals in 4 Croatian counties (Šibenik-Knin, Koprivnica-Križevci, Brod-Posavina, and Varaždin County) were reviewed. According to 2011 Census, the population of these counties comprises 13.1% of the Croatian population.

**Results:**

There were 663 patients with VTE; 408 (61.54%) had deep vein thrombosis, 219 (33.03%) had pulmonary embolism, and 36 (5.43%) had both conditions. Median age was 71 years, 290 (43.7%) were men and 373 (56.3%) women. Secondary VTE was found in 57.3% of participants, idiopathic VTE in 42.7%, and recurrent VTE in 11.9%. There were no differences between patients with secondary VTE and patients with idiopathic VTE in disease recurrence and sex. The most frequent causes of secondary VTE were cancer (40.8%), and trauma, surgery, and immobilization (38.2%), while 42.9% patients with secondary VTE had ≥2 causes. There were 8.9% patients ≤45 years; 3.3% with idiopathic or recurrent VTE. Seventy patients (10.6%) died, more of whom had secondary (81.4%) than idiopathic (18.6%) VTE (*P* < 0.001), and in 50.0% VTE was the main cause of death. Estimated incidence of VTE in Croatia was 1.185 per 1000 people.

**Conclusion:**

Characteristics of VTE in Croatia are similar to those reported in large international studies. Improved thromboprophylaxis during the presence of risk factors for secondary VTE might substantially lower the VTE burden.

Venous thromboembolism (VTE), including deep venous thrombosis (DVT) and pulmonary embolism (PE), is a major health problem in the world, associated with significant morbidity and mortality (1-9). Incidence rates for VTE mostly vary from 1 to 2 in 1000 individuals per year (1-5,7,9). PE, the most serious manifestation of VTE, has a mortality rate of more than 15% in the first 3 months after diagnosis, with short-term survival of less than 60% (10,11). Cohen et al estimated that the number of VTE-related deaths across the European Union (EU) was 543 454 per year, which was more than double the number of combined deaths in EU due to AIDS, breast and prostate cancer, and traffic accidents (8).

VTE in survivors is associated with several chronic consequences of the disease that can severely impair the patients’ quality of life, including post-thrombotic syndrome (PTS) and pulmonary hypertension (PH), as well as recurrent VTE. PTS affects at least one-third of patients after DVT (8,12-15) and PH affects 4%-5% of patients after PE (8,16,17). VTE has significant incidence of recurrence: 10.1% at 6 months, 12.9% after 1 year, and 30.4% after 10 years (18).

Total VTE-related costs to health care system are enormous. For example, the total cost of VTE to the UK National Health Service in 1993 was £235-£257 million (€349-€382 million), and the combined direct and indirect costs in 2004/2005 were approximately £640 million (€950 million), and are even higher when PTS is taken into account (8,19,20).

VTE is a multifactorial disease, resulting from a complex interaction of genetic and acquired factors. Although some studies estimated that genetics was related to up to 60% of the risk of VTE (including FV Leiden and prothrombin G20210A mutations, deficiencies of protein C, S and antithrombin, and elevations of some procoagulant factors) (21), there is also a large number of acquired risk factors for VTE such as immobilization, surgery, trauma, cancer, pregnancy and puerperium, oral contraceptives, autoimmune diseases, and other disorders (1-8,21).

In spite of the importance of VTE, there is not enough data on its incidence and characteristics in transitional countries. Also, although several studies analyzed the epidemiology of VTE in different study settings (1-9), there is still not much information on conditions present at the diagnosis of thrombosis, comparing idiopathic and secondary (provoked) VTE. Therefore, the Croatian Cooperative Group for Hematologic Diseases (CROHEM) analyzed the incidence and characteristics of idiopathic and secondary newly diagnosed VTE in Croatia in 2011, the year of the most recent national population census.

## Material and methods

CROHEM conducted an observational non interventional study of patients with newly diagnosed VTE hospitalized in major general hospitals in four Croatian counties: Šibenik-Knin (General Hospital Šibenik, Šibenik, and General Hospital “Hrvatski Ponos,” Knin), Koprivnica-Križevci (General Hospital “Dr. Tomislav Bardek,” Koprivnica), Brod-Posavina (General Hospital “Dr. Josip Benčević” in Slavonski Brod and Nova Gradiška), and Varaždin County (General Hospital Varaždin) from January 1 until December 31 2011. The study was approved by the medical ethics committee of each hospital.

From patients’ hospital records we collected information on age, sex, date of incident, category of VTE (DVT, PE, or both); characteristics of VTE (idiopathic or secondary, first or recurrent); and comorbidities. Diagnosis and treatment of VTE was performed in each hospital according to standard clinical practice and local policy and was not part of this study analysis.

VTE was classified as idiopathic or secondary. It was classified as secondary VTE (DVT, PE, or both) if it was associated with major trauma, surgery, or marked immobility; sepsis; cancer active within 6 months of VTE event; implanted venous catheter; pregnancy or puerperium at the time of VTE; usage of some prothrombotic medicaments at the time of VTE (ie, oral contraceptives, hormonal replacement therapy); autoimmune or other prothrombotic diseases (ie, antiphospholipid syndrome, systemic lupus erythematosus, inflammatory bowel disease, neurologic diseases with paralysis); or if occurred after >6 h long distance airplane flight. VTE was classified as idiopathic when none of these precipitating factors were registered. VTE was also classified as the first or recurrent. VTE was considered recurrent if prior VTE events were registered in the medical record. Patients who died during hospitalization when VTE was diagnosed were recorded and the main cause of death was categorized as VTE-related or related to some other main cause.

According to the 2011 Population Census, the Republic of Croatia had 4 284 889 inhabitants (48.2% men), and the population was on average 41.7 years old (men 39.9, women 43.4 years), which places it among the oldest nations in Europe (22). Of the counties included in this study, one was from the southern (Dalmatian) part of Croatia (Šibenik-Knin County, with 109 375 inhabitants, 53 596 [49%] men, average age 44.1 years), two were from the northern part of Croatia (Varaždin County, with 175 951 inhabitants, 85 645 [48.7%] men, average age 41.2 years, and Koprivnica-Križevci County, with 115 584 inhabitants, 55 964 [48.4%] men, average age 41.6 years), and one from the eastern (Slavonian) part of Croatia (Brod-Posavina County, with 158 574 inhabitants, 77 115 [48.6%] men, average age 40.6 years). These 4 counties have 559 485 inhabitants (272 320 (48.7%) men), which represents 13.1% of Croatian population (22).

### Statistical analysis

Categorical data are presented with absolute (N) and relative (%) frequencies and compared using χ^2^-test, and numerical data are presented with median and range values and compared using *t* test. Only *P* < 0.050 was considered significant. Annual incidence per 1000 inhabitants was calculated using the number of recorded new VTE cases as the numerator and the population at risk in the 4 counties as the denominator, according to the 2011 Census (22). Data were analyzed using MedCalc program (MedCalc Statistical Software version 15.6.1, MedCalc Software bvba, Ostend, Belgium).

## Results

In 2011, there were 663 new cases of VTE in 4 Croatian counties: 408 (61.54%) of them had DVT, 219 (33.03%) had PE, and 36 (5.43%) had both conditions (Table 1). The majority of patients were elderly people (median age 71 years, range 13-97 years), and there were more women (N = 373, 56.3%) (Table 1). Female patients were significantly older (median 74, range 21-97 years) than male (median 65, range 13-90 years; *P* < 0.001). Patients with PE or with both PE and DVT were significantly older than patients with DVT alone (median and range; 74 [21-97] vs 69 [13-92] years, *P* = 0.006).

**Table 1:** Diagnosis, sex, and age of patients with newly diagnosed venous thromboembolism (VTE) in 4 Croatian counties in 2011*

County	Total VTE (N)	Diagnosis VTE N (% VTE)			Sex		Age in years (median, range)
		DVT	PE	DVT+PE	male N (%)	female N (%)	
**Šibenik-Knin**	151	99 (65.56)	43 (28.46)	9 (5.96)	64 (42.4)	87 (57.62)	72 (13-92)
**Koprivnica-Križevci**	87	54 (62.07)	26 (29.89)	7 (8.05)	39 (44.8)	48 (55.2)	69 (26-90)
**Varaždin**	298	159 (53.36)	128 (42.95)	11 (3.69)	125 (41.9)	173 (58.1)	73 (21-97)
**Brod-Posavina**	127	96 (75.59)	22 (17.32)	9 (7.09)	62 (48.82)	65 (51.2)	67 (33-88)
**Total**	663	408 (61.54)	219 (33.03)	36 (5.43)	290 (43.7)	373 (56.3)	71 (13-97)

*PE – pulmonary embolism; DVT – deep venous thrombosis.

Recurrent VTE was diagnosed in 79 (11.9%) patients (Table 2). There were more patients with secondary (N = 380, 57.3%) than with idiopathic VTE (N = 283, 42.7%) (Tables 2-3). Patients with idiopathic VTE were significantly older than those with secondary VTE (median and range; 74 [26-92] vs 69 [13-97] years, *P* = 0.001). There were no differences between patients with secondary and patients with idiopathic VTE in disease recurrence and sex.

**Table 2:** Idiopathic and recurrent venous thromboembolism (VTE), death, and cause of death of patients with newly diagnosed VTE in 4 Croatian counties in 2011

County	Idiopathic N (%)	Reccurent N (%)	Death N (% VTE)	Reason of death N (% of death)	
				VTE	other diseases
**Šibenik-Knin**	74 (49.0)	18 (11.9)	11 (7.29)	4 (36.4)	7 (63.6)
**Koprivnica-Križevci**	40 (46.0)	10 (11.5)	6 (6.9)	4 (66.7)	2 (33.3)
**Varaždin**	122 (40.9)	39 (13.1)	38 (12.8)	16 (42.1)	22 (57.9)
**Brod-Posavina**	47 (37.0)	12 (9.5)	15 (11.8)	11 (73.3)	4 (26.7)
**Total**	283 (42.7)	79 (11.9)	70 (10.6)	35 (50.0)	35 (50.0)

**Table 3:** Causes of secondary newly diagnosed venous thromboembolism (VTE) in 4 Croatian counties in 2011

County	Secondary N (% all VTE in county and total)	Causes of secondary VTE* (N, % of secondary VTE in county and total)		
		cancer	trauma, surgery, immobilization	other^†^
**Šibenik-Knin**	77 (51.0)	39 (50.7)	23 (29.9)	28 (36.4)
**Koprivnica-Križevci**	47 (54.0)	20 (42.6)	24 (51.1)	19 (40.4)
**Varaždin**	176 (59.06)	68 (38.6)	61 (34.7)	87 (49.4)
**Brod-Posavina**	80 (63.0)	28 (22.1)	37 (46.3)	45 (56.3)
**Total**	380 (57.3)	155 (40.8)	145 (38.2)	179 (47.1)

*163 (42.9%) secondary VTE had >1 cause of secondary VTE.

†Other causes of VTE included other diseases (eg, autoimmune diseases, inflammatory bowel diseases, neurological diseases with paralysis), sepsis, central venous catheter, drugs, pregnancy and puerperium, >6 h airplane flight.

The most frequent causes of secondary VTE were cancer (40.8% of secondary VTE) and trauma, surgery, and immobilization (38.2% of secondary VTE). Other causes included sepsis, other diseases (eg, autoimmune diseases, inflammatory bowel diseases, neurological diseases with paralysis), central venous catheter, drugs, pregnancy and puerperium, and >6 h airplane flight. Patients with trauma, surgery, or immobilization were significantly younger than other patients with VTE (median and range; 67 [13-90] vs 72 [21-97] years, *P* = 0.003). Many of the 380 cases of secondary VTE (N = 163, 42.9% of secondary VTE) had more than one underlying condition (Table 3).

There were 59 (8.9%) VTE patients aged 45 or younger (Table 4). Among them there were significantly more men than women (59.3% vs 40.7%, *P* = 0.011), significantly more cases of secondary than of idiopathic VTE (11.6% vs 5.3%, *P* = 0.005), and 22 cases (3.3% of all VTE patients) of idiopathic or recurrent VTE. There was no significant difference in VTE incidence between seasons or months of the year (data not shown).

**Table 4:** Patients aged 45 years or younger with newly diagnosed venous thromboembolism (VTE) in 4 Croatian counties in 2011

County	Younger ≤45 years (N, % of all VTE in county and total)			
**total**	**idiopathic**	**recurrent**	**idiopathic or recurrent**
**Šibenik-Knin**	16 (10.6)	3 (1.9)	3 (1.9)	6 (3.9)
**Koprivnica-Križevci**	9 (10.3)	1 (1.15)	2 (2.3)	3 (3.45)
**Varaždin**	22 (7.4)	8 (2.7)	2 (0.7)	10 (3.4)
**Brod-Posavina**	12 (9.5)	3 (2.4)	1 (0.8)	3 (2.4)
**Total**	59 (8.9)	15 (2.3)	8 (1.2)	22 (3.3)

Among all 663 VTE patients, 70 (10.6%) died, 50.0% of them with VTE as the main cause of death (Table 2). There was no difference in sex and no difference in VTE recurrence between patients who died and those who survived. Patients who died were significantly older than those who survived (median and range; 77 [26-90] vs 70 [13-97] years, *P* = 0.001). Among patients who died, significantly more patients had secondary (81.4%) than idiopathic (18.6%) VTE (*P* < 0.001).

When we compared the counties according to the type of VTE (DVT, PE, or both), Varaždin County had the highest percentage and Brod-Posavina County the lowest percentage of PE (*P* < 0.001). Furthermore, Brod-Posavina County had the highest percentage and Varaždin County the lowest percentage of DVT (*P* < 0.001, Table 1). There were no significant differences between counties in age and sex of the patients, recurrence of VTE, percentage of idiopathic or secondary VTE, mortality, and cause of death. When we compared the counties according to the cause of secondary VTE, Brod-Posavina County had the highest percentage of trauma, surgery and immobilization (29.1%, *P* = 0.020), and Varaždin County the highest percentage of sepsis (9.4%, *P* < 0.001).

Varaždin County (1.694 per 1000 persons) and Šibenik-Knin County (1.381 per 1000 persons) had significantly higher incidence of VTE than Brod-Posavina County (0.8 per 1000 persons) and Koprivnica-Križevci County (0.753 per 1000 persons; *P* < 0.001, Table 5). Estimated annual incidence of VTE in Croatia according to these 4 counties was 1.185 per 1000 people.

**Table 5:** Incidence of venous thromboembolism (VTE) in 4 Croatian counties and estimated VTE incidence for the entire Croatia in 2011

County	Number of inhabitants in 2011 (N, % of whole Croatia)	VTE (N)	Incidence of VTE per 1000 inhabitants
Šibenik-Knin*	109,375 (2.6)	151	1.381
**Koprivnica-Križevci**	115,584 (2.7)	87	0.753
Varaždin*	175,951 (4.1)	298	1.694
**Brod-Posavina**	158,575 (3.7)	127	0.800
**Total**	559,485 (13.1)	663	1.185

*Counties with higher incidence of VTE than other counties, *P* < 0.001.

## Discussion

Our study showed that the estimated incidence of VTE in Croatia in 2011 was 1.185 per 1000 people. This estimate, as well as the characteristics of VTE observed in this study, are similar to the findings of large international studies, although there are also some differences. Age is an important risk factor for VTE. Several studies showed that the incidence of VTE markedly increased with increasing age for both sexes, from fewer than 5 first-time VTE cases per 100 000 per year among children younger than 15 years to 450-600 cases per 100 000 per year among people older than 80 years (1,3,5). Indeed, in our study the majority of patients were older people (median age 71 years). In the past 50 years, the average age in Croatia increased by almost 10 years (from 32.5 in 1961 to 41.7 years in 2011), mostly due to a continuous decrease in fertility and increase in life expectancy (22).

In our study, patients with PE or with both PE and DVT were older than patients with DVT alone. Other studies showed that the elderly more often had PE and had more serious VTE than younger patients, and that in their case massive PE was particularly life-threatening (23).

Although other studies mostly found no consistent differences in the incidence of VTE between men and women (5), our study found more female (56.3%) than male (43.7%) patients with newly diagnosed VTE. In Croatia there are more women (51.8%) than men (48.2%), but there are also different proportions of men and women in different age groups. For example, there are more men in younger age groups and more women in older age groups, starting with the age group 45-49 years (22). In our study, which mostly included elderly patients, female patients were significantly older than male, but in the subgroup of patients aged 45 years or younger there were more male patients, reflecting the age-sex structure in Croatia.

In our study, recurrent VTE was diagnosed in 11.9% of patients. Recurrent VTE has been already recognized as a serious problem. For example, in a prospective cohort study of 355 patients with DVT, Prandoni et al found recurrent VTE in 8.6% of cases after 6 months and in 30.3% of cases after 8 years (12), while Heit et al reported recurrent VTE in 10.1% of cases after 6 months, in 12.9% after 1 year, and in 30.4% after 10 years (18).

VTE is a multifactorial disease resulting from a complex interaction between different inherited (genetic) and acquired prothrombotic factors. Inherited thrombophilia is a predisposition to thrombosis and exposes carriers to increased risks for VTE compared with non-carriers. Coen et al showed that the prevalence of FV Leiden and prothrombin G20210A mutations were higher in Croatian patients with VTE than in healthy subjects (24), and Jukic et al confirmed the association of non-00 blood group genotypes with increased risk of VTE in Croatia (25). However, it is still a matter of intense debate under which circumstances comprehensive and expensive laboratory genetic testing for inherited thrombophilia is useful in clinical practice. Some authors suggest that thrombophilia screening should be considered in patients with VTE aged 45 years or younger, especially in those with idiopathic or recurrent VTE (26). Therefore, we analyzed the patients aged 45 years or younger and found 59 (8.9%) patients in that subgroup, with more cases of secondary than idiopathic VTE, and 22 of them (just 3.3% of all VTE patients) had idiopathic or recurrent VTE, representing possible candidates for genetic thrombophila testing.

In our study, there were more patients with secondary than with idiopathic VTE. In other studies, the proportion of patients with idiopathic VTE was between 26% and 47% of first-time cases, partly depending on the definition of idiopathic and secondary VTE used in the studies (5). In our study patients with idiopathic VTE were significantly older than those with secondary VTE, although both groups included mostly elderly patients. There was no difference in disease recurrence or sex between patients with secondary and idiopathic VTE. The most frequent cause of secondary VTE was cancer (40.8% of secondary VTE), but 42.9% of cases with secondary VTE had more than one underlying condition associated with VTE.

Cancer is one of the strongest risk factors for VTE and, vice versa, VTE is a frequent complication of malignancy and may be the earliest manifestation of an occult cancer (27). The association of cancer and VTE is due to multiple factors: malignant cells can produce hypercoagulable state through multiple mechanisms, and the risk for VTE is further increased by chemotherapy and other drugs for cancer treatment, the use of central venous catheters, surgery, and prolonged immobility (28,29). These patients require appropriate thromboprophylaxis and adequate long term anticoagulant treatment if VTE occurs (30).

The second most frequent cause of secondary VTE in our study was trauma, surgery, and immobilization (38.2% of secondary VTE). It is known that surgery is related to a 6-fold increased risk of VTE (7,9). The 9th edition of Antithrombotic Therapy and Prevention of Thrombosis Guidelines of the American College of Chest Physicians state that the high incidence of postoperative VTE mandates that thromboprophylaxis should be considered in every surgical patient (31).

Although some studies showed a higher incidence of fatal PE during winter, others did not confirm this finding (5). Similarly, we did not find a significant difference in VTE incidence either between seasons or between the months of the year.

In our study, 70 (10.6%) VTE patients died and in 50% of them VTE was the main cause of death. Patients who died and those who survived did not differ in sex and recurrence of VTE. The literature reports that the risk of dying is highest shortly after the VTE event, and that during the first year after the VTE it gradually approaches that in the general population (7). In our study, patients who died were significantly older than those who survived. Among patients who died, there were more patients with secondary than with idiopathic VTE. In patients with cancer, VTE is the second leading cause of death (32), and cancer was the most frequent underlying condition for secondary VTE in our study.

Diagnosis and treatment of VTE was performed in each hospital according to the standard clinical practice and local policy. Therefore, the decision to perform Color Doppler ultrasound analysis of peripheral veins to exclude DVT in patients with confirmed PE was made by the local physician depending of the clinical presentation and local policy, and was not part of this study. This might explain the finding that 219 (33.03%) patients had PE alone and just 36 (5.43%) patients had confirmed both PE and DVT. However, some studies also reported a small percentage (6%) of patients with both PE and DVT (7) and most clinical studies that did not include autopsy data described the incidence of clinically diagnosed DVT to be approximately twice that of PE (5), which is similar to our finding.

Varaždin County and Šibenik-Knin County had higher incidence of VTE than Brod-Posavina County and Koprivnica-Križevci County. These differences might be explained by the fact that Šibenik-Knin County has the oldest population among the studied counties (average age 44.1 years) and is the second oldest county in Croatia after Lika-Senj County (22). On the other hand, General Hospital Varaždin, the only general hospital in Varaždin County, admits many chronically ill patients with immobility transferred from other regional special hospitals for chronic diseases or from rehabilitation centers, who are at a greater risk for VTE event (personal communication).

The estimated annual incidence of VTE in our study was 1.185 per 1000 people (or 118.5 per 100 000 people), which is comparable to other literature data, where VTE incidence rates varied from 1 to 2 per 1000 individuals per year (1-5,7,9). The epidemiology of VTE is always challenging: beside real differences, differences between studies may also arise from differences in study design, patients cohorts, and case definition (7).

This study has some limitations. First, the true incidence of VTE may have been underestimated for several reasons: we reviewed inpatient medical records and did not include VTE cases treated outside of the county general hospitals (for example cases from nursing homes or sudden deaths from community). However, outpatient treatment of newly DVT and especially PE is extremely rare in Croatia. It is also likely that there were fatal and/or non diagnosed VTE events that were not inluded in this analysis. Another potential limitation is that assessment of associated conditions and classification as idiopathic or secondary VTE were limited by contents of the medical record, and some data were missing for many patients such as smoking, obesity, diet, and family history for VTE.

Strengths of this study include involvement of 4 different counties from different parts of Croatia, representing 13.1% of Croatian population according to the national population Census that was done in the same year as this study, thus representing the most accurate data. We believe that our study made a reliable estimate of the burden of VTE in Croatia, describing its characteristics and associated substantial mortality, which might help policy-makers to develop strategies dealing with this major health problem.

However, further research is needed to estimate VTE incidence in Croatia more accurately, to describe trends in incidence over time, and to implement optimal VTE prevention and management. The literature data showed that the type, duration, and intensity of prophylaxis were frequently inappropriate and suboptimal (33-35). In our study, the most common risk factors for secondary VTE were cancer, trauma, surgery, and immobilization, and many cases of secondary VTE had more than one underlying condition. Since VTE is a preventable disease and effective prophylaxis is widely available – in addition to the development of new anticoagulants (36) – improved thromboprophylaxis in these settings might substantially lower the incidence and mortality of VTE.
